# Proximate Composition, Physicochemical Properties and Concentration of Selected Minerals in Edible Giblets of Geese

**DOI:** 10.3390/foods14152742

**Published:** 2025-08-06

**Authors:** Dariusz Kokoszyński, Arkadiusz Nędzarek, Joanna Żochowska-Kujawska, Marek Kotowicz, Marcin Wegner, Karol Włodarczyk, Dorota Cygan-Szczegielniak, Barbara Biesiada-Drzazga, Marcin Witkowski

**Affiliations:** 1 Department of Animal Breeding and Nutrition, Bydgoszcz University of Science and Technology, 85084 Bydgoszcz, Poland; karwlo19@gmail.com; 2 Department of Aquatic Bioengineering and Aquaculture, Faculty of Food Sciences and Fisheries, West Pomeranian University of Technology in Szczecin, 71-550 Szczecin, Poland; arkadiusz.nedzarek@zut.edu.pl; 3 Department of Meat Science, West Pomeranian University of Technology, 71-550 Szczecin, Poland; joanna.zochowska-kujawska@zut.edu.pl (J.Ż.-K.); marek.kotowicz@zut.edu.pl (M.K.); marcinwit97@gmail.com (M.W.); 4 Boehringer-Ingelheim, 00-728 Warsaw, Poland; marcin.wegner@op.pl; 5 Department of Biology and Animal Environment, Bydgoszcz University of Science and Technology, 85-084 Bydgoszcz, Poland; dorota.cygan-szczegielniak@pbs.edu.pl; 6 Institute of Animal Science and Fisheries, University of Siedlce, 08-110 Siedlce, Poland; barbara.drzazga@wp.pl

**Keywords:** geese, breed, sex, edible giblets, chemical composition, minerals, meat color, electrical conductivity, acidity

## Abstract

The purpose of this study was to determine the effect of breed and sex (3 × 2) on the basic chemical composition, concentration of some minerals, and physicochemical properties of edible giblets of farm geese. The study material consisted of edible giblets (livers, gizzards, hearts) obtained from 42 geese from three Polish native breeds (Rypin, Suwałki, Kartuzy) at 220 weeks of age. Edible giblets were obtained during goose evisceration from seven males and seven females of each breed. Each bird was an experimental unit. Goose breed and sex had a significant effect on the chemical composition and physicochemical properties of the edible giblets. Rypin geese had higher (*p* < 0.05) intramuscular fat content in the gizzard and heart, as well as higher protein content in the heart and lower water content in the gizzard, compared to Kartuzy and Suwałki geese. Kartuzy geese, in turn, had higher content of water in the heart, and higher concentrations of phosphorus, calcium, iron, manganese, sodium, and chromium in the liver, compared to Rypin and Suwałki geese. In turn, Suwałki geese had higher concentrations of phosphorus in the gizzard, and potassium, phosphorus, copper, and iron in the heart compared to the hearts of Rypin and Suwałki geese, while Kartuzy and Suwałki geese higher concentrations of sodium, magnesium, zinc, and manganese in hearts than the hearts of Rypin geese. In these studies, the highest lightness (L*) was observed in the liver and heart of Rypin geese, the lowest yellowness (b*) was observed in the gizzard of Suwałki geese, and the highest pH_24_ and EC_24_ were observed in the heart of Kartuzy geese. Regardless of breed, males had higher protein, collagen, and intramuscular fat contents in the heart, a higher water content in the gizzard, higher concentrations of potassium, and sodium in the liver and gizzard, copper in the heart and liver, and phosphorus in the gizzard, and less water in the heart and zinc in the liver, as well as higher (*p* < 0.05) concentrations of iron in the liver and heart compared with females. The breed by sex interaction was significant for intramuscular fat and water content in the gizzard and heart, and protein content in the heart. Significant differences were also noted for EC_24_ in the liver and heart, yellowness of the gizzard, and concentrations of most labeled minerals in edible giblets. The obtained results indicate that the nutritional value and suitability of edible goose giblets for the poultry industry vary depending on breed and sex. Due to the limited research on the chemical composition and physicochemical properties of goose giblets, further research in this area is necessary in the future.

## 1. Introduction

Goose meat production in 2023 was 5.21 million tons, representing 3.6% of the total poultry meat. China was the largest producer of goose meat at that time, supplying 97.6% (5.09 million tons) of global production. In Europe, the leader in goose meat production in recent years has been Poland, alternating with Hungary [1].

In recent decades, raw materials obtained from native goose breeds have become increasingly popular with consumers due to their high nutritional value and sensory qualities [2]. Currently, 10 domestic and 4 foreign goose breeds in Poland are covered by the Genetic Resources Conservation Programme for Goose, and these goose breeds are also classified by the FAO as global genetic resources of domesticated goose breeds subject to protection. Their preservation is important due to the need to maintain genetic biodiversity and preserve traditional meat production systems, their high suitability for backyard and organic farming, and the functional properties of their meat. Native goose breeds are also important as a source of self-supply for rural areas in meat and feathers, as well as in the production of traditional regional products [3,4,5,6,7].

Edible poultry giblets include livers, gizzards, and hearts [8]. The yield of goose giblets is around 5.2–7.3% [5]. Poultry giblets are packaged and sold together with carcasses, especially duck and goose carcasses, or are distributed as separate raw materials (livers, gizzards, hearts) for further processing or retail sale [9]. Today, edible giblets are ingredients in many traditional dishes and poultry preparations in the cuisines of many countries around the world. Among the most popular are homogenous offal sausages (liver sausage, liverwurst sausage), foie gras, offal preserves (stews, paprikash, poultry livers in fat), and snack food in Asia [4,6,10,11,12]. Among edible poultry giblets, liver is the most popular among consumers due to its high nutritional value, low price, delectable taste, and quick and easy cooking [11,13,14]. Liver contains high-quality protein, iron, copper, and zinc, and is a good source of vitamins B_2_, B_9_, B_12_, A, and D [11,15,16]. In contrast, gizzard is abundant in protein, iron, and zinc, while poultry heart is rich in fat [17,18].

In the available scientific literature, there are relatively few studies on poultry giblets [8,19,20,21,22,23,24], especially geese [19,24]. In a study by Nagy et al. [19] conducted on Egyptian goose species, it was shown that among edible giblets, liver had the highest protein content (24.80%). The gizzard had the highest moisture content (72.42%), while the heart had the highest fat content (12.18%). In this study [19], moreover, gizzard meat had the highest acidity (pH = 6.43), while liver had the lowest acidity (pH = 6.72). Wojtycza et al. [24], on the other hand, reported significantly higher amounts of Fe and Cu in 100 g of liver from 16-week-old White Kołuda^®^ males than the daily human requirement for these minerals.

Poultry offal, including livers, hearts, and gizzards, is a crucial component of the meat industry, both for economic and nutritional reasons. Goose offal, in particular, plays a significant role in the animal products market, especially in countries with a strong goose-farming tradition, such as Poland, Hungary, and France. Interest in this product category stems from its favorable nutritional profile-rich in protein, heme iron, B vitamins, and trace elements—making it a valuable component of the human diet [25,26]. Furthermore, offal, being a valuable source of trace elements, usually provides larger amounts of these components than meat [9,27]. Therefore, for economic reasons, these products can be sold commercially as important components in human and animal nutrition. When incorporated into many animal products, they affect the nutritional value of the final product [27].

Although, in recent years, there has been a trend of decline in giblet consumption in many European countries, nutritionists draw attention to the high nutritional and dietary value and the possibility of diversifying the human diet by encouraging their consumption of offal [16,28]. From a scientific point of view, analysis of the chemical composition and nutritional value of goose offal provides the data necessary to assess their suitability for human nutrition and the production of functional foods. Studies have shown that goose liver, also known as foie gras, is characterized by a high fat content, as well as valuable unsaturated fatty acids and fat-soluble vitamins [29]. In turn, hearts and gizzards, as striated muscles, contain significant amounts of high-quality protein and are a source of minerals such as zinc and selenium [30]. In addition to studies on poultry offal [9], offal from other animals also showed a more favorable mineral profile compared to meat [27]. Research by Biel et al. [27] showed that offal from veal, beef, and lamb had higher concentrations of Zn, Fe, Cu, Mn, Ca, and Na compared to thigh. Due to the health consequences of deficiencies of these elements, conducting research in this area is of great scientific importance, especially if it concerns less common species. Iron and zinc deficiencies can cause anemia, rickets, and impaired cognitive and immune functions [31], while deficiencies of Se, Cu, Zn, Fe, and Mn can impair the function of key enzymes that neutralize free radicals in the body [27,31]. Therefore, providing these elements through the diet is crucial.

From an economic perspective, the use of offal as a valuable food product contributes to increased profitability in poultry production and reduced post-production losses, thus supporting the concept of sustainable agriculture and a circular economy [32]. However, there is still a lack of sufficient data on the detailed nutritional profile of offal from geese compared to other poultry species, justifying the need for further research in this area.

Also, the available scientific literature did not find information on the influence of Polish native goose breed and gender on the basic chemical composition, mineral concentration, and physicochemical characteristics of edible giblets. In addition, so far, the offal of Polish native geese has not been evaluated, and the values of electrical conductivity and collagen content in the offal of different poultry species have not been determined, which provides an additional impetus for the implementation of this study. The results obtained in this study supplement the knowledge in this area.

The purpose of this research was to determine the proximate chemical composition (protein, intramuscular fat, collagen, water content), physicochemical properties (acidity, electrical conductivity, L*, a*, b* color variables), and concentration of ten selected minerals in edible giblets of geese.

## 2. Materials and Methods

### 2.1. Research Material

The study material consisted of edible giblets (livers, gizzards, hearts) of 42 geese from three Polish native goose breeds (Rypin, Suwałki, Kartuzy). Edible giblets were obtained from 7 male and 7 female geese of each breed, 14 birds of each breed, at 220 weeks of age, after four reproductive seasons. The experiment was conducted in a 3-breed x 2-sex design. Edible offal was obtained individually from each goose. Each bird was an experimental unit. The slaughter date of the examined breeds of geese was related to the date of liquidation of breeding goose breeds, and was determined by the provisions of the Genetic Resources Conservation Programme for Geese implemented in Poland. According to the breeder, the studied goose breeds were kept in the same indoor building during the reproductive period. Air temperature ranged from 5 to 25 °C depending on the month, and relative humidity was 60–70%. The birds were housed in 12 pens (4 pens for each breed), each measuring 80 m^2^, with a stocking density of 0.9 birds/m^2^. The male-to-female ratio was 1:3.5. Daylight duration during the reproductive period was 10 h/day. Yellow LED lighting was used. The birds were fed a complete diet in the form of industrial commercial goose feed mixtures *ad libitum*. The feed mixture for the reproductive period contained 14.8% protein, 2.3% oils and fat, and 4.8% crude fiber. The birds were transported for slaughter in a specialized poultry transport vehicle. The geese were slaughtered as part of regular agricultural practices at a commercial poultry slaughterhouse according to the regulations laid down by the Polish poultry industry. Ethical approval was not required for this study according to Polish legal regulations (The Act of the Polish Parliament of 15 January 2015 on the Protection of Animals Used for Scientific or Educational Purposes, Journal of Laws 2015, item 266). The giblets obtained during evisceration were transported for 2 h (in a car refrigerator) to the laboratory of the university and then stored for 22 h after delivery to the laboratory at 4°C in a refrigerated cabinet (Hendi, Robakowo, Poland). The cooled giblets were subjected to evaluation. The determination of pH_24_, EC_24_, and color parameters L*, a*, b* was performed 24 h after goose slaughter, approximate composition was assessed on fresh samples after determining physicochemical properties, and the samples for determining minerals were frozen and stored at 18 °C until their freeze-drying (approximately 3 months).

### 2.2. Proximate Composition

Proximate composition (protein, collagen, intramuscular fat, water content) was determined using a Food Scan™ 2 Meat Analyser (Food Scan, Hillerød, Denmark). Measurements were made by near-infrared (NIR) transmission spectrometry using calibration on artificial neural nets (ANN) according to PN-A-82109 [33].

### 2.3. Minerals

Macronutrients, including phosphorus (P), potassium (K), sodium (Na), magnesium (Mg), and calcium (Ca), and microelements, including iron (Fe), copper (Cu), zinc (Zn), magnesium (Mn), and chromium (Cr), were assessed in freeze-dried samples of edible giblets: livers, gizzards, and hearts. Freeze-drying of the samples was carried out in an Alpha 1–4 LD plus dryer (Christ, Osterode, Germany) at minus 50 °C, at a pressure of 0.63 atm for 24 h until a solid mass was obtained. Then freeze-dried samples of 0.5 ± 0.001 g each were dissolved by the wet method with 6 mL of a mixture of nitric acid HNO_3_ and perchloric acid HClO_4_ (5:1 ratio). The dissolved samples were cooled to room temperature and then diluted with Milli-Q water (18.2 MΩ) to a volume of 25 mL. Mineral concentrations in samples of edible giblets (livers, gizzards, hearts) were determined using ZA300 atomic absorption spectrophotometry (Hitachi, Tokyo, Japan). Calcium, potassium, sodium, and magnesium were determined by acetylene/air flame atomization (FAAS). On the other hand, iron, copper, zinc, manganese, and chromium concentrations were measured by graphite cuvette atomization under an argon atmosphere (GFAAS). Phosphorus (P) content, on the other hand, was determined by a colorimetric method using a UV-VIS U-2900 spectrophotometer (Hitachi, Tokyo, Japan).

### 2.4. Physicochemical Analysis

Acidity (pH_24_), electrical conductivity (EC_24_), and color variables (in the CIELab system) were determined after cooling edible giblets, 24 h after slaughter. A CX-701 Multifunction meter (Elmetron, Zabrze, Poland) equipped with a dagger electrode was used to measure the acidity of edible giblet meat. An LF-Star CPU conductivity meter (Ingenierbürno R. Matthäus, Norbitz, Germany) was used to measure electrical conductivity. The meat color parameters (L*—lightness; a*—redness; b*—yellowness) were measured with a CR-400 chroma meter (Konica Minolta, Chiyoda-ku, Japan). The measurement area was about 0.5 cm^2^, the measurement aperture had a diameter of 8 mm, and the illumination was D_65_. The chroma meter was calibrated on calibration plates before taking L*a*b* variable measurements.

### 2.5. Statistical Analysis

The data collected during this study on proximate chemical composition, concentrations of some minerals, and physicochemical characteristics were statistically analyzed. The effects of breed (3 levels), sex (2 levels), and their interactions (breed x sex) were analyzed with a general linear model (GLM) in SAS 9.4. [34]. For each trait, the arithmetic mean and standard deviation were calculated, as well as the standard error of the mean. The significance of differences at *p* < 0.05 between the arithmetic means of the studied traits of geese was assessed using Tukey’s test.

## 3. Results and Discussion

### 3.1. Proximate Chemical Composition

The goose breeds compared differed (*p* < 0.05) in the percentage of intramuscular fat and water in the gizzard, as well as in protein, intramuscular fat, and water contents in the heart. Rypin geese had significantly higher (*p* < 0.05) percentages of protein and intramuscular fat in the heart compared to Suwałki and Kartuzy geese. In contrast, the water content of the gizzard of Rypin geese was significantly lower (*p* < 0.05) compared to the percentage of water in the gizzards of Suwałki and Kartuzy geese. The sex of the birds significantly affected the percentage of water in the gizzard and the percentage of protein, collagen, intramuscular fat, and water in the hearts. Regardless of breed, males had a significantly (*p* < 0.05) higher percentage of water in the gizzard, and protein, collagen, and intramuscular fat in hearts than females. In contrast, female geese had a significantly higher percentage of water in their hearts than males (Table 1). A study by Nagy et al. [19] found higher water and protein content in the gizzard and heart of Egyptian goose species compared to the results of the present study conducted on edible giblets of Polish native breeds of geese. The goose breeds (Rypin, Suwałki, Kartuzy) compared in our study had higher fat content in the gizzard than gizzards obtained from Egyptian goose species [19] and lower fat content in the heart than hearts of Kartuzy and Suwałki geese. Abdullah and Buchtová [20] showed lower protein and fat content but higher water content in the gizzard and heart of 45-day-old commercial crossbred Cherry Valley ducks. Studies conducted on chickens [9] revealed higher water content and lower protein content in the gizzard and heart of chickens compared to the studied goose breeds.

### 3.2. Minerals

Comparing the studied breeds of geese in terms of the concentration of some minerals in the liver, there was significant variation in the compared Polish native breeds of geese in terms of the concentration of labeled minerals, except potassium and magnesium. Significantly (*p* < 0.05) higher concentrations of macroelements (P—phosphorus; Na—sodium; Ca—calcium) and microelements (Fe—iron; Cu—copper; Mn—manganese; Cr—chromium) were found in the liver of Kartuzy geese compared to the livers of Rypin and Suwalki geese. The exception was the concentration of zinc in the liver; the highest concentration of this metal was found in the liver of Rypin geese. The sex of the birds significantly differentiated the studied birds in terms of K, Na, Fe, Cu, and Zn concentrations. The statistically significantly higher levels of potassium, sodium, iron, and copper in the liver of males (Table 2), obtained in this study, may result from the physiologically higher activity of oxidative enzymes in these individuals compared to females. The cofactors of these enzymes are the aforementioned elements [31,35]. Research by Ren et al. [35] showed that zinc supplementation improves metabolic and antioxidant functions in goose liver. Biochemically, dietary copper binds to albumin and histidine and is then transported to the liver via the portal circulation, forming complexes with cytoplasmic proteins in the cells of this organ. After ingestion, zinc accumulates mainly in the liver and is then distributed throughout the body, hence the highest content of these elements in the liver compared to other organs [31]. The intensity of these transformations and the related difference in the content of minerals in tissues may vary depending on sex, age, breed, or health status of the animals [9]. This could have caused significant variation in the content of certain elements in offal between individual breeds of Polish geese (Rypin, Suwałki, Kartuzy). In turn, the statistically significantly higher levels of sodium and potassium in the liver of males obtained in this study may be related to more intense energy metabolism in males. In the study by Biel et al. [27], the liver of male cattle and calves also contained higher levels of Fe, Zn, Cu, and K, which could be due to metabolic and hormonal differences occurring physiologically between the sexes. Furthermore, the liver, heart, and stomach are organs with different structures, so the metabolic activity itself will vary depending on the specifications and function of these organs [27,35,36].

The interaction of breed versus sex was significant (*p* = 0.001 to 0.017) for concentrations of phosphorus, potassium, sodium, magnesium, iron, zinc, and chrome in liver (Table 2). A study by Wojtyczka et al. [24] found higher concentrations of P, Mg, Na, K, Cu, Fe, and Zn, but lower amounts of calcium in the livers of 17-week-old White Kołuda^®^ males (pedigree strain W-33) compared to the ratio of the aforementioned elements in the livers of the Rypin, Suwalki, and Kartuzy geese studied. On the other hand, Ciślik et al. [37] determined generally lower concentrations of Mn and Zn, but lower concentrations of Cu in the livers of the Zatory goose breed than in the livers of the goose breeds tested. Jokanović et al. [9] evaluated edible giblets (livers, gizzards, and hearts) of chickens obtained lower concentrations of P, Fe, Cu, Zn, and Mn, while higher concentrations of Mg and K (per 100 g tissue) were found in livers compared to the livers of Polish native geese tested after four reproductive seasons. On the other hand, Seong et al. [18] found higher concentrations of phosphorus (P) in the livers of broiler chickens, except for Kartuzy geese in this study, as well as higher concentrations of K, Na, and Mg and lower concentrations of Ca, Fe, Cu, Mn and Zn compared to the livers of aged geese in this study. Majewska et al. [11] compared mineral concentrations in the livers of African ostriches, turkeys, and broiler chickens and found the highest concentrations of phosphorus, potassium, and magnesium in turkey livers. African ostrich livers, however, were characterized by high calcium, iron, and zinc contents.

Analyzing the three geese breeds for macro- and micronutrient concentrations in the gizzard, significant differences were found in terms of phosphorus, calcium, iron, manganese, and chromium concentrations. Kartuzy geese had significantly higher concentrations of calcium, iron, manganese, and chromium in the gizzard compared to Rypin and Suwałki geese. Suwałki geese had the highest concentration of phosphorus in the gizzard. The interaction between breed and sex was significant for sodium, copper, manganese, and chromium concentrations in the gizzard (Table 3). A study by Jokanović et al. [9] conducted on broiler chickens found lower concentrations of P, Na, Ca, Fe, Cu, Zn, and Mn, while typically higher concentrations of K were found in the gizzard compared to the gizzards in this study of geese after four reproductive seasons. Bucław et al. [8] found higher concentrations of K, Zn, and Ca, but lower concentrations of P, Mg, Ca, Fe, and Cu in the gizzards of 15-year-old male and female emus than in the 5-year-old geese in this study. The higher concentrations of phosphorus, potassium, and sodium in male stomachs (Table 3), obtained in our study, may result from differences in metabolic activity and the intensity of mechanical digestion in the gizzard. In the study by Kheravia et al. [38], male broilers (Ross 308) fed a high-sodium diet showed greater gizzard development and lower pH, which promoted better mineral absorption and could increase sodium and potassium transport into stomach cells. Males may also have physiologically larger stomachs compared to females, which may determine these differences. The content of mineral salts, especially iron, zinc, and copper, is particularly high in organs rich in myoglobin and oxidative enzymes, such as the liver and heart. Heme iron is found primarily in myoglobin and cytochromes, the levels of which depend on the intensity of cellular respiration. High iron and zinc contents in goose offal were documented in a study by Okruszek et al. [30], and their concentrations may be modulated by genetic factors related to the breed, growth rate, and metabolic capacity of the bird [39].

Analysis of the geese studied in terms of the concentration of some minerals in the heart showed significant differences (*p* < 0.05) in the concentration of labeled minerals, except for calcium and chromium. Suwałki geese had significantly higher concentrations of potassium, phosphorus, iron, and copper in the heart than the hearts of Rypin and Kartuzy geese. Suwałki and Kartuzy geese were significantly superior to Rypin geese in the concentration of sodium, magnesium, zinc, and manganese in hearts. Males had significantly higher concentrations of Fe (*p* < 0.05) and Cu (*p* = 0.002) in hearts than females. The interaction between breed and sex was statistically significant only for iron concentration (Table 4). A study on 15-year-old male and female emus [8] reported higher concentrations of P, K, Mg, and Zn, but lower concentrations of Ca, Mn, and Cr in 100 g of hearts from these birds compared to hearts from Rypin, Kartuzy, and Suwałki geese in this study.

### 3.3. Physical and Chemical Properties

The compared goose breeds were also evaluated in terms of physicochemical traits (acidity pH_24_, electrical conductivity EC_24_, color parameters L*, a*, b*) of edible giblets Table 5, Table 6 and Table 7. Rypin geese had significantly higher L* *(p* = 0.002) and yellowness (b* = 0.039) of livers compared to Suwalki and Kartuzy geese. Regardless of breed, the livers of male geese were significantly (*p* < 0.05) darker in color (lower L* and b*) than females. The interaction of breed vs. sex was significant for EC_24_ of the liver. In this study, there was a significant effect of breed on the yellowness (b*) of the gizzard, and there were significant differences in L* and a* of the gizzard between males and females, and a significant breed vs. sex interaction for b* (yellowness) of the liver. The results of the physicochemical traits of the heart meat of the geese studied indicated a significant effect of breed on acidity (pH_24_) and electrical conductivity (EC_24_). Significant differences were found between males and females for pH_24_ and EC_24_. The interaction between breed and sex was significant for heart EC_24_. Abdullah and Buchtová [20] found higher liver lightness and yellowness values, as well as higher L* and b* and lower a* of hearts of 45-day-old Cherry Valley ducks compared to the geese studied in this study. The pH of livers, gizzards, and hearts obtained in our study was lower than the pH of the same organs in the Egyptian goose species [19].

**Table 5:** Physicochemical properties of the liver of aged geese.

Trait		Breed			Sex		SEM	*p*-Value		
		Rypin (*n* = 14)	Suwalki (*n* = 14)	Kartuzy (*n* = 14)	Male (*n* = 21)	Female (*n* = 21)		Breed	Sex	Interaction
pH_24_—acidity	mean sd	6.02 0.14	5.88 6.13	6.02 0.26	6.00 0.14	5.94 0.23	0.03	0.097	0.292	0.752
EC_24_—conductivity (mS/cm)	mean sd	8.01 2.19	6.50 2.63	7.20 1.55	6.95 2.53	7.53 1.84	0.34	0.072	0.272	<0.001
L*—lightness	mean sd	40.76 ^a^ 2.95	38.52 ^a^ 6.40	33.79 ^b^ 5.37	35.75 6.20	39.63 * 4.74	0.89	0.002	0.014	0.633
a*—redness	mean sd	13.95 1.90	13.83 2.80	13.28 3.17	13.30 2.69	14.07 2.57	0.40	0.778	0.353	0.349
b*—yellowness	mean sd	8.01 ^a^ 3.06	6.52 ^b^ 3.35	5.12 ^b^ 2.73	5.30 2.87	7.79 * 3.11	0.49	0.039	0.008	0.766

^a, b^—Mean values of traits in rows marked with different letters are statistically significantly different (*p* < 0.05). *—Statistically significant difference between males and females (*p* < 0.05). SEM—standard error of the mean.

**Table 6:** Physicochemical properties of the gizzard of aged geese.

Trait		Breed			Sex		SEM	*p*-Value		
		Rypin (*n* = 14)	Suwalki (*n* = 14)	Kartuzy (*n* = 14)	Male (*n* = 21)	Female (*n* = 21)		Breed	Sex	Interaction
pH_24_—acidity	mean sd	6.20 0.40	6.30 0.15	6.38 0.20	6.33 0.36	6.25 0.15	0.04	0.230	0.401	0.519
EC_24_—conductivity (mS/cm)	mean sd	6.27 1.65	6.21 2.14	7.48 2.03	6.28 2.00	7.02 1.96	0.30	0.169	0.228	0.883
L*—lightness	mean sd	33.74 2.94	34.10 2.57	37.22 9.08	33.34 2.62	36.70 * 7.47	0.89	0.182	0.049	0.142
a*—redness	mean sd	13.95 3.54	14.44 2.84	14.95 3.31	15.64 2.20	13.26 * 3.62	0.50	0.698	0.017	0.649
b*—redness	mean sd	3.15 ^a^ 3.90	1.68 ^b^ 0.73	4.78 ^a^ 4.03	2.97 3.30	3.44 3.62	0.53	0.034	0.618	0.014

^a, b^—Mean values of traits in rows marked with different letters are statistically significantly different (*p* < 0.05). *—Statistically significant difference between males and females (*p* < 0.05). SEM—standard error of the mean.

**Table 7:** Physicochemical properties of the heart of aged geese.

Trait		Breed			Sex		SEM	*p*-Value		
		Rypin (*n* = 14)	Suwalki (*n* = 14)	Kartuzy (*n* = 14)	Male (*n* = 21)	Female (*n* = 21)		Breed	Sex	Interaction
pH_24_—acidity	mean sd	6.13 ^b^ 0.11	6.09 ^b^ 0.13	6.27 ^a^ 0.17	6.20 0.14	6.13 * 0.17	0.02	0.002	0.048	0.054
EC_24_—conductivity (mS/cm)	mean sd	5.89 ^a^ 2.55	4.78 ^b^ 1.04	5.99 ^a^ 1.19	6.16 1.93	4.94 * 1.41	0.27	0.018	0.002	<0.001
L*—lightness	mean sd	34.02 ^a^ 3.30	29.66 ^b^ 3.18	31.01 ^b^ 5.00	31.23 4.01	31.89 4.53	0.65	0.020	0.601	0.574
a*—redness	mean sd	19.12 3.90	17.90 2.86	17.42 5.27	18.45 4.62	17.85 3.57	0.63	0.559	0.651	0.868
b*—redness	mean sd	3.61 1.60	2.33 1.17	3.25 2.04	2.64 1.55	3.49 1.75	0.26	0.115	0.103	0874

^a, b^—Mean values of traits in rows marked with different letters are statistically significantly different (*p* < 0.05). *—Statistically significant difference between males and females (*p* < 0.05). SEM—standard error of the mean.

The nutritional value and physicochemical properties of offal, such as hearts, livers, and gizzards of slaughter birds, are influenced by a number of interrelated biochemical and genetic factors. Among the key parameters determining the quality of these tissues is enzymatic activity and the intensity of tissue metabolism, which varies depending on the organ’s function and the metabolic rate characteristic for a given species [40,41]. The pH of offal, in turn, reflects postmortem glycolysis, where muscle glycogen is converted to lactic acid. The rate and extent of pH decline after slaughter vary among tissue types and are regulated by both enzymatic activity and the degree of perfusion of the organ [42]. It is believed that avian livers and hearts have a higher final pH than skeletal muscle, which may influence their susceptibility to microbial growth, as well as their color and texture. The color of offal, an important parameter of sensory quality, depends primarily on the content of myoglobin and its chemical forms (oxymyoglobin, metmyoglobin, deoxymyoglobin). Genotypic differences in the expression of proteins responsible for oxygen metabolism affect color hue and durability. Geese and ducks, compared to chickens, have a higher content of color pigments, which translates into a darker liver and heart color [25,43]. The influence of nutritional and environmental factors, such as the level of micronutrients in the diet and pre-slaughter stress conditions, which modify both the mineral profile and the physicochemical properties of internal organs, is also significant [29,44].

## 4. Conclusions

In summary, after four reproductive seasons, three Polish native breeds of geese (Kartuzy, Rypin, Suwałki) differed in terms of intramuscular fat and water content in gizzards and hearts, and protein content in hearts. The compared goose breeds differed significantly in terms of the concentration of most of the determined minerals. The goose breed had a significant effect on the acidity and conductivity of the heart and the lightness of the liver and heart, and the yellowness of the liver and gizzard. The sex of the birds significantly affected the proximate chemical composition of the heart and the water content in the gizzard, as well as the concentration of most minerals, except for the concentration of macroelements in hearts and microelements in gizzards. Kartuzy geese had the lowest intramuscular fat content in the gizzard and heart and higher concentrations of many minerals, including the highest iron, calcium, and manganese content in the gizzard and liver, which may indicate their higher nutritional value compared to Suwałki and Rypin geese. This study provided information on the chemical composition and physicochemical characteristics of edible giblets of Polish native goose breeds that may be useful for consumers and processors of these raw materials, indicating their nutritional value and suitability for the poultry industry. Future research should expand to include sensory assessment, including instrumental evaluation of textural characteristics and microstructural characteristics of goose giblets.

## Figures and Tables

**Table 1:** Basic chemical composition of the gizzard and heart of aged geese.

Trait		Breed			Sex		SEM	*p*-Value		
		Rypin (*n* = 14)	Suwalki (*n* = 14)	Kartuzy (*n* = 14)	Male (*n* = 21)	Female (*n* = 21)		Breed	Sex	Interaction
Gizzard										
Protein (%)	mean sd	18.76 0.29	19.08 1.08	18.77 0.48	18.85 0.98	18.89 0.19	0.11	0.441	0.843	0.171
Collagen (%)	mean sd	2.63 0.26	2.66 0.30	2.85 0.30	2.65 0.31	2.79 0.27	0.04	0.141	0.140	0.365
Intramuscular fat (%)	mean sd	5.00 ^a^ 1.40	4.37 ^a^ 1.84	2.56 ^b^ 0.75	3.84 2.05	4.11 1.36	0.26	<0.001	0.499	0.001
Water (%)	mean sd	68.93 ^b^ 0.75	69.57 ^a^ 1.28	70.70 ^a^ 1.81	70.57 1.64	68.89 * 0.71	0.23	<0.001	<0.001	<0.001
Heart										
Protein (%)	mean sd	19.16 ^a^ 2.84	18.93 ^b^ 1.94	18.90 ^b^ 2.06	21.07 0.51	16.92 * 0.30	0.33	0.008	<0.001	<0.001
Collagen (%)	mean sd	1.74 0.62	1.74 0.48	1.85 0.42	2.20 0.14	1.36 * 0.28	0.07	0.585	<0.001	0.131
Intramuscular fat (%)	mean sd	12.66 ^a^ 0.87	11.81 ^b^ 0.24	11.17 ^b^ 0.19	12.18 1.04	11.58 * 0.28	0.12	<0.001	<0.001	<0.001
Water (%)	mean sd	56.85 ^b^ 1.55	57.55 ^b^ 1.11	58.83 ^a^ 1.84	56.40 0.78	59.09 * 1.09	0.26	<0.001	<0.001	0.007

^a, b^—Mean values of traits in rows marked with different letters are statistically significantly different (*p* < 0.05). *—Statistically significant difference between males and females (*p* < 0.05). SEM—standard error of the mean.

**Table 2:** Content of selected minerals in the liver of aged geese.

Trait		Breed			Sex		SEM	*p*-Value		
		Rypin (*n* = 14)	Suwalki (*n* = 14)	Kartuzy (*n* = 14)	Male (*n* = 21)	Female (*n* = 21)		Breed	Sex	Interaction
Macroelements (mg/100 g of liver)										
Phosphorus	mean sd	278.67 ^b^ 28.86	292.24 ^b^ 49.71	312.27 ^a^ 39.49	295.74 44.91	293.04 39.25	6.43	0.018	0.772	<0.001
Potassium	mean sd	182.98 18.85	178.62 29.69	195.61 23.34	196.16 25.60	175.32 * 19.59	3.84	0.070	0.001	0.004
Sodium	mean sd	80.43 ^b^ 8.42	82.73 ^b^ 15.32	97.53 ^a^ 10.70	91.21 15.03	82.59 * 11.34	2.13	<0.001	0.005	0.001
Magnesium	mean sd	18.30 1.32	18.39 2.79	19.48 1.89	19.18 2.18	18.26 1.97	0.32	0.128	0.081	0.001
Calcium	mean sd	12.58 ^b^ 3.87	9.29 ^b^ 2.16	22.43 ^a^ 8.78	13.75 7.76	15.79 8.11	1.22	<0.001	0.249	0.322
Microelements (mg/100 g of liver)										
Iron	mean sd	20.81 ^b^ 2.26	21.09 ^b^ 4.16	26.93 ^a^ 6.06	25.48 5.13	20.40 * 3.93	0.80	<0.001	<0.001	0.017
Copper	mean	11.00 ^b^	11.24 ^b^	31.46 ^a^	22.76	13.04 *	2.07	<0.001	0.001	0.480
	sd	6.36	6.51	13.69	14.63	10.27				
Zinc	mean	3.97 ^a^	3.37 ^b^	3.91 ^a^	3.57	3.93 *	0.01	0.007	0.032	0.001
	sd	0.96	0.49	0.42	0.51	0.84				
Manganese	mean	0.29 ^b^	0.27 ^b^	0.32 ^a^	0.30	0.29	0.01	0.003	0.165	0.005
	sd	0.02	0.04	0.04	0.03	0.01				
Chrome	mean	0.03 ^b^	0.02 ^b^	0.04 ^a^	0.03	0.03	0.01	0.001	0.058	0.159
	sd	0.01	0.01	0.01	0.01	0.01				

^a, b^—Mean values of traits in rows marked with different letters are statistically significantly different (*p* < 0.05). *—Statistically significant difference between males and females (*p* < 0.05). SEM—standard error of the mean.

**Table 3:** Content of selected minerals in the gizzard of aged geese.

Trait		Breed			Sex		SEM	*p*-Value		
		Rypin (*n* = 14)	Suwalki (*n* = 14)	Kartuzy (*n* = 14)	Male (*n* = 21)	Female (*n* = 21)		Breed	Sex	Interaction
Macroelements (mg/100 g of gizzard)										
Phosphorus	mean sd	140.02 ^b^ 8.83	147.81 ^a^ 15.51	133.65 ^b^ 11.47	145.14 12.86	135.84 * 12.34	2.05	0.007	0.011	0.151
Potassium	mean sd	183.48 17.89	195.39 18.10	188.61 16.24	196.53 14.68	181.78 * 17.63	2.72	0.163	0.005	0.848
Sodium	mean sd	77.00 9.25	84.04 11.25	81.63 6.06	83.42 9.36	78.42 * 8.86	1.44	0.071	0.045	0.004
Magnesium	mean sd	16.43 1.60	16.73 1.53	15.91 1.19	16.57 1.25	16.14 1.64	0.22	0.344	0.364	0.776
Calcium	mean sd	33.06 ^b^ 5.83	28.20 ^b^ 7.56	51.65 ^a^ 20.64	39.22 18.89	36.06 13.73	2.57	<0.001	0.439	0.293
Microelements (mg/100 g of gizzard)										
Iron	mean sd	4.47 ^b^ 0.54	5.14 ^b^ 1.83	6.57 ^a^ 1.36	5.63 1.82	5.15 1.33	0.24	0.001	0.262	0.614
Zinc	mean	3.06	3.12	3.27	3.11	3.05	0.01	0.851	0.580	0.887
	sd	0.28	0.39	8.69	10.18	9.84				
Copper	mean	0.39	0.35	0.40	0.40	0.35	0.02	0.372	0.094	0.001
	sd	0.14	0.08	0.12	0.12	0.10				
Manganese	mean	0.08 ^b^	0.08 ^b^	0.19 ^a^	0.11	0.12	0.05	<0.001	0.066	0.017
	sd	0.01	0.02	0.04	0.05	0.07				
Chrome	mean	0.04 ^b^	0.09 ^b^	0.16 ^a^	0.11	0.08	0.01	<0.001	0.170	0.049
	sd	0.01	0.05	0.06	0.07	0.06				

^a, b^—Mean values of traits in rows marked with different letters are statistically significantly different (*p* < 0.05). *—Statistically significant difference between males and females (*p* < 0.05), SEM—standard error of the mean.

**Table 4:** Content of selected minerals in the heart of aged geese.

Trait		Breed			Sex		SEM	*p*-Value		
		Rypin (*n* = 14)	Suwalki (*n* = 14)	Kartuzy (*n* = 14)	Male (*n* = 21)	Female (*n* = 21)		Breed	Sex	Interaction
Macroelements (mg/100 g of heart)										
Potassium	mean sd	144.81 ^b^ 10.93	183.18 ^a^ 18.53	158.54 ^b^ 6.37	162.97 21.73	161.38 19.76	3.15	<0.001	0.753	0.965
Phosphorus	mean sd	184.09 ^b^ 17.05	225.71 ^a^ 29.03	200.08 ^b^ 12.84	205.93 29.71	200.06 21.80	0.35	0.001	0.513	0.883
Sodium	mean sd	94.04 ^b^ 7.49	117.40 ^a^ 12.13	108.50 ^a^ 4.59	107.68 15.08	105.61 10.63	1.98	<0.001	0.533	0.702
Magnesium	mean sd	17.00 ^b^ 1.24	20.94 ^a^ 2.16	18.91 ^a^ 1.17	19.08 2.44	18.81 2.13	0.35	<0.001	0.656	0.886
Calcium	mean sd	27.07 7.74	23.38 2.28	24.60 7.56	24.86 6.82	25.17 6.08	0.97	0.463	0.899	0.730
Microelements (mg/100 g of heart)										
Iron	mean sd	7.59 ^b^ 0.73	10.85 ^a^ 2.33	8.67 ^b^ 1.03	9.71 2.47	8.37 * 1.17	0.31	<0.001	0.006	0.016
Zinc	mean	2.34 ^b^	2.85 ^a^	2.60 ^a^	2.61	2.59	0.05	0.001	0.815	0.169
	sd	0.21	0.35	0.19	0.38	0.27				
Copper	mean	0.97 ^b^	1.52 ^a^	1.13 ^b^	1.38	1.03 *	0.06	0.006	0.002	0.614
	sd	0.23	0.34	0.39	0.38	0.32				
Manganese	mean	0.09 ^b^	0.11 ^a^	0.11 ^a^	0.11	0.10	0.01	0.004	0.342	0.525
	sd	0.01	0.02	0.01	0.02	0.01				
Chrome	mean	0.03	0.02	0.03	0.02	0.03	0.01	0.331	0.603	0.501
	sd	0.02	0.01	0.02	0.02	0.03				

^a, b^—Mean values of traits in rows marked with different letters are statistically significantly different (*p* < 0.05). *—Statistically significant difference between males and females (*p* < 0.05), SEM—standard error of the mean.

## Data Availability

The original contributions presented in the study are included in the article, further inquiries can be directed to the corresponding author.
